# Circulating MicroRNAs Reflect Systemic Inflammation and Metabolic Dysfunction in Oesophageal Squamous Cell Carcinoma–Associated Cachexia

**DOI:** 10.1002/jcsm.70409

**Published:** 2026-10-22

**Authors:** Yan Li, Rui Chen, Xinyi Lv, Shujuan Wei, Yiming Xu, Shengguang Ding

**Affiliations:** ^1^ Department of Cardiovascular Surgery Southeast University Affiliated Nantong First People's Hospital Nantong China; ^2^ Department of Pathology Nantong University Nantong China; ^3^ Department of Clinical Medicine Nantong University Nantong China; ^4^ Department of Thoracic Surgery Southeast University Affiliated Nantong First People's Hospital Nantong China

**Keywords:** biomarkers, cancer cachexia, ESCC, miRNAs, systemic inflammation

## Abstract

**Background:**

Cachexia in oesophageal squamous cell carcinoma is driven by systemic inflammation and malnutrition and is associated with poor survival. We evaluated whether circulating miRNAs can identify ESCC cachexia and reflect its inflammatory‐nutritional phenotype.

**Methods:**

We enrolled 126 participants: 42 healthy controls and 84 treatment‐naïve patients with ESCC, equally classified as noncachectic or cachectic using international consensus criteria. Small‐RNA sequencing in a discovery subset (*n* = 4/group) was followed by cohort‐wide qRT‐PCR. Exploratory within‐cohort discrimination was assessed by receiver operating characteristic (ROC) analysis; survival associations were evaluated using Kaplan–Meier and univariate Cox analyses.

**Results:**

Cachectic ESCC was characterized by lower albumin, total protein and prealbumin and higher IL‐6, TNF‐α, CRP and IL‐1β than noncachectic ESCC (all *p* < 0.05). Circulating hsa‐miR‐5187‐5p and hsa‐miR‐4433b‐3p increased stepwise from controls to noncachectic and cachectic ESCC. For noncachectic versus cachectic ESCC, AUCs were 0.8594 and 0.8175, respectively (both *p* < 0.0001); for controls versus cachectic ESCC, AUCs were 0.9439 and 0.8889. Cachexia was associated with shorter overall survival (HR 1.708, 95% CI 1.011–2.887; *p* = 0.026), as were high miR‐5187‐5p (HR 3.635) and miR‐4433b‐3p (HR 3.190) expression. Correlations with inflammatory markers and albumin were weak to moderate.

**Conclusions:**

hsa‐miR‐5187‐5p and hsa‐miR‐4433b‐3p are candidate circulating indicators of ESCC‐associated cachexia and its inflammatory‐metabolic milieu. Their discriminatory and prognostic performance remains exploratory because analyses were conducted within the same cohort and were not comprehensively adjusted for tumour stage or other clinicopathological factors.

## 1 Introduction

Oesophageal cancer remains a leading cause of cancer‐related mortality worldwide and is frequently accompanied by profound metabolic and nutritional disturbances. Oesophageal squamous cell carcinoma (ESCC) accounts for approximately 90% of cases and carries a poor prognosis, with a 5‐year survival rate of ~20% [1, 2]. Cancer cachexia is a multifactorial syndrome characterized by progressive skeletal muscle loss, with or without adipose tissue depletion, that cannot be fully reversed by conventional nutritional support and leads to functional impairment [3]. In ESCC, reduced oral intake, early satiety and treatment‐related gastrointestinal toxicity may amplify the effects of tumour‐driven systemic inflammation and metabolic catabolism [2, 4, 5, 6]. Cachexia is clinically important because it is associated with poorer treatment tolerance, postoperative complications, reduced quality of life and shorter survival [7, 8, 9]. Although international consensus criteria standardize diagnosis [3], routine assessment largely depends on established phenotypic changes such as weight loss and low BMI. Objective blood‐based biomarkers could therefore complement clinical assessment and potentially facilitate risk stratification and longitudinal monitoring.

Circulating microRNAs (miRNAs) are small noncoding RNAs that regulate gene expression posttranscriptionally and participate in inflammation, metabolism, differentiation, apoptosis and tissue remodelling [10]. They circulate in relatively stable forms within extracellular vesicles or bound to proteins and lipoproteins, allowing reproducible detection in plasma or serum [11, 12, 13]. Circulating miRNA signatures have been investigated as diagnostic and prognostic biomarkers across cancers, including ESCC, where several miRNAs are associated with tumour presence, stage, metastatic burden and survival [14, 15]. Most ESCC studies, however, have focused on tumour diagnosis or prognosis rather than cachexia as a systemic host phenotype.

There is also biological rationale for studying miRNAs in cachexia. Experimental studies indicate that tumour‐derived extracellular‐vesicle miRNAs can modulate skeletal muscle biology; cancer‐derived miR‐21, for example, can promote myoblast apoptosis through innate immune signalling [16]. MiRNAs linked to inflammatory signalling, myogenesis, mitochondrial function and proteolysis have therefore been proposed as candidate markers or mediators of cancer cachexia [17]. This is particularly relevant to ESCC, in which reduced intake may coexist with systemic inflammation and metabolic dysfunction [6, 18]. However, to date, comprehensive clinical studies specifically examining circulating miRNA profiles in ESCC‐associated cachexia are lacking.

We therefore integrated clinical cachexia phenotyping with plasma miRNA profiling to identify circulating miRNAs associated with ESCC‐related cachexia. We further examined their relationships with inflammatory cytokines, CRP, albumin and survival; explored within‐cohort discrimination of cachectic versus noncachectic disease; and used GO/KEGG and predicted target networks to identify potentially relevant pathways. Because circulating miRNA measurements are sensitive to sample processing, haemolysis, RNA isolation and normalization [19, 20] and because this study did not include an independent validation cohort, biomarker performance analyses were prespecified as exploratory.

## 2 Methods

### 2.1 Human Participants and Clinical Sample Collection

A total of 126 participants were enrolled at Nantong First People's Hospital, including 42 healthy volunteers and 84 patients with histopathologically confirmed ESCC. Newly diagnosed patients were consecutively recruited between January and December 2021 and had received no radiotherapy, chemotherapy or other antitumour treatment before surgery. ESCC patients were classified as cachectic (*n* = 42) or noncachectic (*n* = 42) according to international consensus criteria [3]. Tumour stage was assigned using the eighth edition UICC/AJCC TNM classification. Patients were excluded for oesophageal adenocarcinoma, gastric cardia carcinoma, active infection, concurrent malignancy, prior antitumour therapy, hormone therapy or long‐term corticosteroid use. Healthy controls were recruited from the hospital health examination centre and had no recent unintended weight loss, major systemic disease or relevant medication use. The study was approved by the Ethics Committee of Nantong First People's Hospital, and all participants provided written informed consent in accordance with the Declaration of Helsinki.

### 2.2 Anthropometric Measurements and Estimated Indices

Height was measured to the nearest centimetre and weight to the nearest 0.5 kg. BMI was calculated as weight/height^2^. Body fat percentage was estimated from BMI and age using sex‐specific equations: 1.2 × BMI + 0.23 × age−16.2 for males and 1.2 × BMI + 0.23 × age−5.4 for females. Estimated fat mass was calculated as body weight × body fat percentage/100, with fat mass index obtained by dividing by height^2^. Estimated fat‐free mass was calculated as body weight × (1 − body fat percentage/100), and fat‐free mass index was calculated similarly. Because these indices were equation‐based rather than directly measured, they were used only for descriptive characterization and were not interpreted as direct measures of skeletal muscle mass or adipose tissue.

### 2.3 miRNA Sequencing and Bioinformatic Analysis

For discovery profiling, plasma small‐RNA sequencing was performed in a subset of 12 participants (*n* = 4 per group), followed by cohort‐wide qRT‐PCR assessment of selected candidates. Sequencing and downstream bioinformatic analyses were performed by Shanghai Biotechnology Corporation. Total RNA was isolated using the miRNeasy Plasma Midi Kit (Qiagen, Cat. No. 77044); small‐RNA libraries were prepared with the QIAseq miRNA Library Kit (Qiagen, Cat. No. 1103679) and sequenced on an Illumina HiSeq platform. Differentially expressed miRNAs were defined by *p* < 0.05 and |log2 fold change| > 2. Expression patterns were visualized using hierarchical clustering heatmaps and volcano plots. GO and KEGG enrichment analyses were performed using the complete sets of differentially expressed miRNAs in ESCC versus healthy controls and cachectic versus noncachectic ESCC. Predicted miRNA‐mRNA‐pathway networks were constructed for hsa‐miR‐5187‐5p and hsa‐miR‐4433b‐3p. Bioinformatic analyses and visualization were performed in R.

### 2.4 Plasma miRNA Extraction, Reverse Transcription and qRT‐PCR

Peripheral blood was centrifuged at 4°C and 4000×*g* for 15 min to obtain plasma. Total miRNA was extracted with the miRcute Plasma miRNA Extraction Kit (TIANGEN, Cat. No. DP503), dissolved in 20‐μL RNase‐free water and stored at −80°C. Reverse transcription used the miRcute Enhanced miRNA cDNA Synthesis Kit (TIANGEN, Cat. No. KR211) in a 20‐μL reaction at 42°C for 60 min followed by 95°C for 3 min. qRT‐PCR used the miRcute Enhanced miRNA Fluorescent Quantitative Detection Kit (TIANGEN, Cat. No. FP411). Amplification comprised 95°C for 15 min, five preamplification cycles (94°C for 20 s, 65°C for 30 s and 72°C for 34 s) and 45 amplification cycles (94°C for 20 s and 60°C for 34 s), followed by melting‐curve analysis. Relative expression was calculated by the 2−ΔΔCt method with U6 small nuclear RNA as the endogenous control. Assays were performed in triplicate and technical replicates were averaged to generate one value per participant. Amplification efficiencies and primer sequences are provided in Tables S1 and S2. The use of U6 as a single endogenous reference is acknowledged as a methodological limitation.

### 2.5 Enzyme‐Linked Immunosorbent Assay (ELISA)

Plasma concentrations of IL‐6, TNF‐α, IL‐1β and CRP were measured using commercially available ELISA kits (Jiangsu Jingmei Bio‐Tech Co. Ltd) in accordance with the manufacturer's protocols. Technical replicates were averaged before statistical analysis.

### 2.6 Statistical Analysis

Clinical and demographic variables were assessed for normality. Normally distributed data are presented as mean ± SD and nonnormally distributed data as median (IQR). Two‐group comparisons used independent‐sample Student's *t* tests or Mann–Whitney U tests, whereas three‐group comparisons used one‐way ANOVA with appropriate post hoc tests or Kruskal–Wallis tests with post hoc comparisons. Categorical variables were analysed using *χ* ^2^ or Fisher's exact tests. miRNA expression was analysed using the same strategy after calculation of relative expression by 2−ΔΔCt.

ROC curves explored within‐cohort discrimination among healthy controls, noncachectic ESCC and cachectic ESCC. AUCs, sensitivities, specificities and cohort‐derived optimal cut‐offs were calculated. Because candidate identification, cohort‐wide qRT‐PCR and ROC assessment were conducted within the same overall cohort, ROC results were considered exploratory and were not treated as independent validation.

Survival analyses were restricted to patients with ESCC. Kaplan–Meier curves were compared using log‐rank tests. Participants were classified into high‐ and low‐expression groups according to the mean value of each biomarker, and HRs with 95% CIs were estimated using univariate Cox proportional‐hazards models. Multivariable Cox regression incorporating TNM stage, age, sex and other prognostic factors was not performed because of the limited cohort size and concern regarding instability and overfitting. Accordingly, survival analyses were considered unadjusted and exploratory.

Logistic regression evaluated associations between candidate biomarkers and group status. Two exploratory multivariable models were constructed: Model 1 included hsa‐miR‐5187‐5p with IL‐6, TNF‐α, IL‐1β, CRP and albumin; Model 2 substituted hsa‐miR‐4433b‐3p. These covariates represented the principal biological domains investigated: miRNA dysregulation, systemic inflammation and biochemical nutritional impairment. Age, sex, TNM stage and other clinicopathological variables were not systematically incorporated into the ROC, correlation, survival or multivariable logistic‐regression analyses. Thus, observed associations cannot be interpreted as independent of tumour stage or overall disease burden. Analyses were performed using SPSS 23.0, R and GraphPad Prism 9.3.1. Tests were two‐sided, with *p* < 0.05 considered significant.

## 3 Results

### 3.1 Clinical and Biochemical Characteristics of the Study Cohort

Clinical characteristics are summarized in Table 1. Because weight loss and BMI contributed to the consensus criteria used to define cachexia, differences in these variables were expected by design and were interpreted descriptively rather than as independent findings. Equation‐derived body fat percentage, fat mass index and fat‐free mass index were likewise descriptive only and were not interpreted as direct evidence of skeletal muscle or adipose tissue depletion.

**TABLE 1:** Baseline clinical characteristics of the study population.

	Healthy control ( *n* = 42)	Oesophageal squamous cell carcinoma (*n* = 84)	
		Noncachexia (*n* = 42)	Cachexia (*n* = 42)
Gender (male/female)	21/21	27/15	32/10
Age (years) (SD)	65.81 (7.31)	69.76 (6.50) ^a^	70.14 (7.43) ^b^
Time (day) (SD)	1409.07 (272.76)	1013.50 (539.47) ^a^	666.10 (367.26) ^b^ ^,^ ^c^
Vital status, *n* (%)			
Alive	—	17 (40.5)	9 (21.4)
Dead	—	25 (59.5)	33 (78.6)
Disease stage, *n* (%)			
II	—	8 (19.0)	2 (4.8)
III	—	20 (47.6)	6 (14.3)
IVa	—	13 (31.0)	24 (57.1)
IVb	—	1 (2.4)	10 (23.8)
Body weight loss (kg) (SD)	0.48 (1.55)	−0.14 (1.81)	−6.06 (1.55) ^b^ ^,^ ^c^
Body weight loss rate (%) (SD)	0.72 (2.31)	−0.07 (3.08)	−10.17 (2.36) ^b^ ^,^ ^c^
Height (m) (SD)	1.65 (0.08)	1.65 (0.08)	1.67 (0.08)
Body mass index (BMI) (kg/m2) (SD)	25.45 (4.72)	22.35 (3.30) ^a^	19.28 (2.65) ^b^ ^,^ ^c^
Percentage of body fat (PBF) (%) (SD)	34.88 (8.38)	30.52 (7.25) ^a^	25.64 (6.63) ^b^ ^,^ ^c^
Fat mass index (FMI) (kg/m2) (SD)	9.15 (3.66)	6.95 (2.33) ^a^	5.05 (1.80) ^b^ ^,^ ^c^
Fat‐free mass index (FFMI) (kg/m2) (SD)	16.31 (2.18)	15.39 (1.85) ^a^	14.23 (1.53) ^b^ ^,^ ^c^
White blood cell ( 10 ^9^ /L) (SD)	5.82 (2.32)	5.86 (1.62)	5.70 (1.76)
Lymphocyte ( %) (SD)	31.65 (32.81)	25.68 (9.14)	24.14 (10.37)
Haemoglobin (g/L) (SD)	136.57 (15.08)	130.48 (14.02)	123.86 (19.94) ^b^
Platelet ( 10 ^9^ /L) (SD)	197.86 (45.92)	203.12 (61.70)	195.55 (79.41)
Albumin ( g/L) (SD)	42.82 (3.30)	41.57 (3.14)	33.58 (3.80) ^b^ ^,^ ^c^
Total protein ( g/L) (SD)	70.61 (7.94)	70.51 (4.09)	59.40 (3.99) ^b^ ^,^ ^c^
Prealbumin ( g/L) (SD)	264.90 (74.29)	228.86 (73.22) ^a^	146.88 (34.53) ^b^ ^,^ ^c^
Creatinine (μmol/L) (SD)	63.87 (11.68)	70.96 (21.88)	68.07 (23.50)
IL‐6 (ng/L) (SD)	18.52 (0.89)	21.75 (0.99) ^a^	22.83 (1.00) ^b^ ^,^ ^c^
TNF‐α (ng/L) (SD)	398.60 (20.04)	433.30 (21.14) ^a^	483.11 (20.25) ^b^ ^,^ ^c^
CRP (ug/L) (SD)	2137.57 (103.01)	2496.64 (116.06) ^a^	2756.38 (123.03) ^b^ ^,^ ^c^
IL‐1 (pg/mL) (SD)	41.84 (2.06)	45.12 (1.79) ^a^	49.33 (1.91) ^b^ ^,^ ^c^

^a^ *p* < 0.05 (noncachexia compared with control subjects).

^b^ *p* < 0.05 (cachexia compared with control subjects).

^c^ *p* < 0.05 (noncachexia compared with cachexia).

Among variables not used to establish cachexia status, cachectic ESCC patients had lower albumin (33.58 ± 3.80 vs. 41.57 ± 3.14 g/L), total protein (59.40 ± 3.99 vs. 70.51 ± 4.09 g/L), prealbumin (146.88 ± 34.53 vs. 228.86 ± 73.22) and haemoglobin than noncachectic patients (all *p* < 0.05). In parallel, IL‐6 (22.83 ± 1.00 vs. 21.75 ± 0.99 ng/L), TNF‐α (483.11 ± 20.25 vs. 433.30 ± 21.14 ng/L), CRP (2756.38 ± 123.03 vs. 2496.64 ± 116.06 μg/L) and IL‐1β (49.33 ± 1.91 vs. 45.12 ± 1.79 pg/mL) were higher in cachectic than noncachectic ESCC (all *p* < 0.05; Table 1 and Figure S1). Leukocyte count, platelet count and creatinine did not differ significantly among groups. Cachectic patients also had a greater proportion of advanced TNM‐stage disease and shorter overall survival; therefore, survival differences were interpreted as unadjusted associations rather than effects attributable to cachexia alone.

### 3.2 Identification and qRT‐PCR Confirmation of Cachexia‐Associated Circulating miRNAs in ESCC

Plasma miRNA profiling revealed distinct expression patterns among healthy controls, noncachectic ESCC and cachectic ESCC (Figure 1A–D). Intersection analysis of miRNAs upregulated in cachectic ESCC versus both healthy controls and noncachectic ESCC identified two candidates, hsa‐miR‐5187‐5p and hsa‐miR‐4433b‐3p (Figure 1E and Table S3). Cohort‐wide qRT‐PCR confirmed a stepwise increase in both miRNAs from healthy controls to noncachectic ESCC and then to cachectic ESCC, with highest expression in the cachectic group (Figure 1F and Tables S4 and S5). These findings supported their further exploratory evaluation as circulating indicators associated with ESCC cachexia.

**FIGURE 1:**
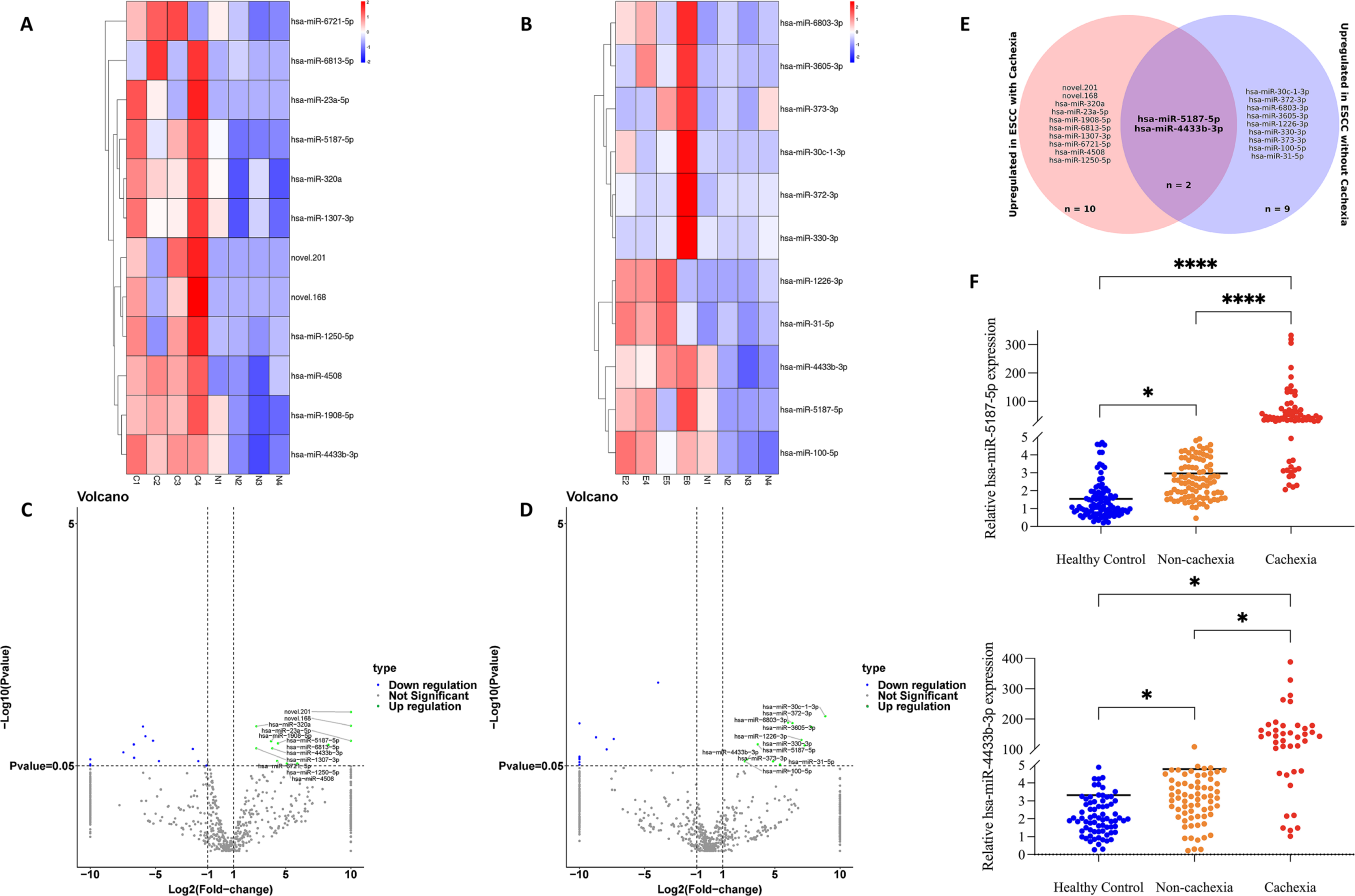
Identification and validation of cachexia‐associated circulating miRNAs in ESCC patients. (A, B) Hierarchical clustering of differentially expressed circulating miRNAs in healthy controls versus cachectic ESCC and cachectic versus noncachectic ESCC. (C, D) Corresponding volcano plots. (E) Intersection of miRNAs upregulated in cachectic ESCC in both comparisons, identifying hsa‐miR‐5187‐5p and hsa‐miR‐4433b‐3p. (F) qRT‐PCR expression in healthy controls, noncachectic ESCC and cachectic ESCC (*n* = 42/group). Points represent participants; bars indicate mean ± SEM. *p < 0.05; ***p < 0.001; ****p < 0.0001.

### 3.3 Exploratory Within‐Cohort ROC Analysis of Circulating miRNAs in ESCC‐Associated Cachexia

ROC analyses were performed in the same overall cohort used for candidate identification and qRT‐PCR assessment (Figure 2). hsa‐miR‐5187‐5p discriminated healthy controls from noncachectic ESCC with an AUC of 0.8435 (95% CI 0.7512–0.9359; *p* < 0.0001; cut‐off > 2.778; sensitivity 97.62%; specificity 71.43%). Discrimination was stronger for healthy controls versus cachectic ESCC (AUC 0.9439, 95% CI 0.8973–0.9904; *p* < 0.0001; cut‐off > 7.032; sensitivity 83.33%; specificity 92.86%) and for noncachectic versus cachectic ESCC (AUC 0.8594, 95% CI 0.7707–0.9481; *p* < 0.0001; cut‐off > 9.523; sensitivity 76.19%; specificity 90.48%).

**FIGURE 2:**
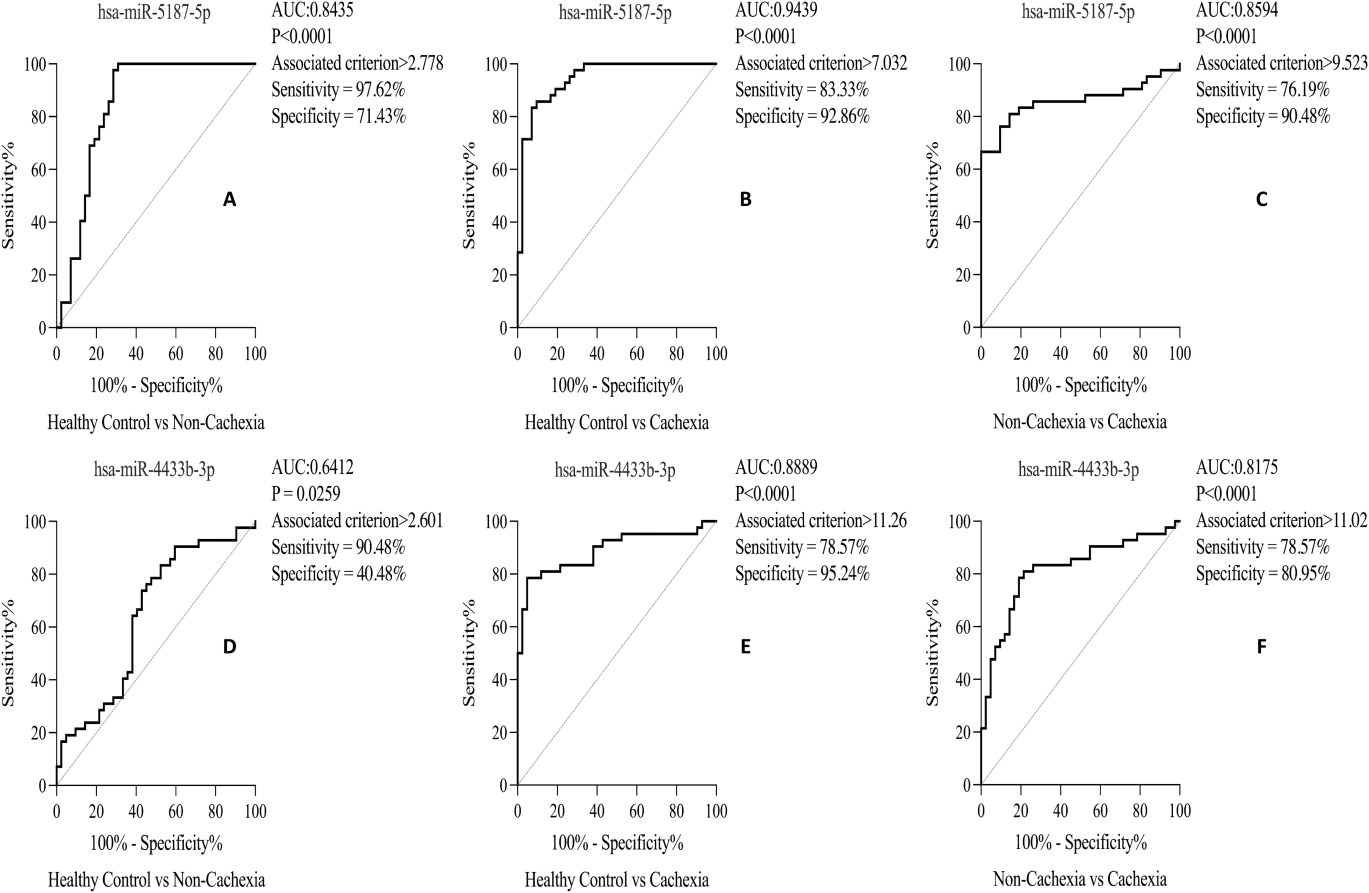
ROC performance of circulating miRNAs for ESCC cachexia classification. ROC curves for hsa‐miR‐5187‐5p (A–C) and hsa‐miR‐4433b‐3p (D–F) comparing healthy controls (HC), noncachectic ESCC (ESCC‐NC) and cachectic ESCC (ESCC‐C). AUC, *p* value, cohort‐derived cut‐off, sensitivity and specificity are shown in each panel.

hsa‐miR‐4433b‐3p showed limited discrimination between healthy controls and noncachectic ESCC (AUC 0.6412, 95% CI 0.5207–0.7616; *p* = 0.0259; cut‐off > 2.601; sensitivity 90.48%; specificity 40.48%) but stronger discrimination for healthy controls versus cachectic ESCC (AUC 0.8889, 95% CI 0.8141–0.9636; *p* < 0.0001; cut‐off > 11.26; sensitivity 78.57%; specificity 95.24%) and noncachectic versus cachectic ESCC (AUC 0.8175, 95% CI 0.7233–0.9116; *p* < 0.0001; cut‐off > 11.02; sensitivity 78.57%; specificity 80.95%). Thus, both candidates showed stronger discrimination in comparisons involving cachectic ESCC. Because discovery, qRT‐PCR and ROC analyses used the same overall cohort, these estimates may be optimistic and require independent validation.

### 3.4 Unadjusted Associations of Cachexia, Circulating miRNAs and Inflammatory Markers With Overall Survival in ESCC

In Kaplan–Meier and univariate Cox analyses, cachectic ESCC was associated with shorter overall survival than noncachectic ESCC (HR 1.708, 95% CI 1.011–2.887; *p* = 0.026; *n* = 42/group; Figure 3A). High hsa‐miR‐5187‐5p expression was associated with shorter survival (HR 3.635, 95% CI 2.113–6.253; *p* < 0.0001; high *n* = 41, low *n* = 42), as was high hsa‐miR‐4433b‐3p (HR 3.190, 95% CI 1.871–5.439; *p* < 0.0001; high *n* = 42, low *n* = 41) (Figure 3B,C). Higher inflammatory markers were also associated with shorter survival: IL‐6 (HR 3.945, 95% CI 1.405–11.08; *p* = 0.0097), TNF‐α (HR 2.152, 95% CI 1.157–4.004; *p* = 0.0037), CRP (HR 3.861, 95% CI 1.954–7.627; *p* = 0.011) and IL‐1β (HR 2.074, 95% CI 1.188–3.622; *p* = 0.002) (Figure 3D–G). These analyses were not adjusted for TNM stage, age, sex or other prognostic factors and therefore do not establish independent prognostic value.

**FIGURE 3:**
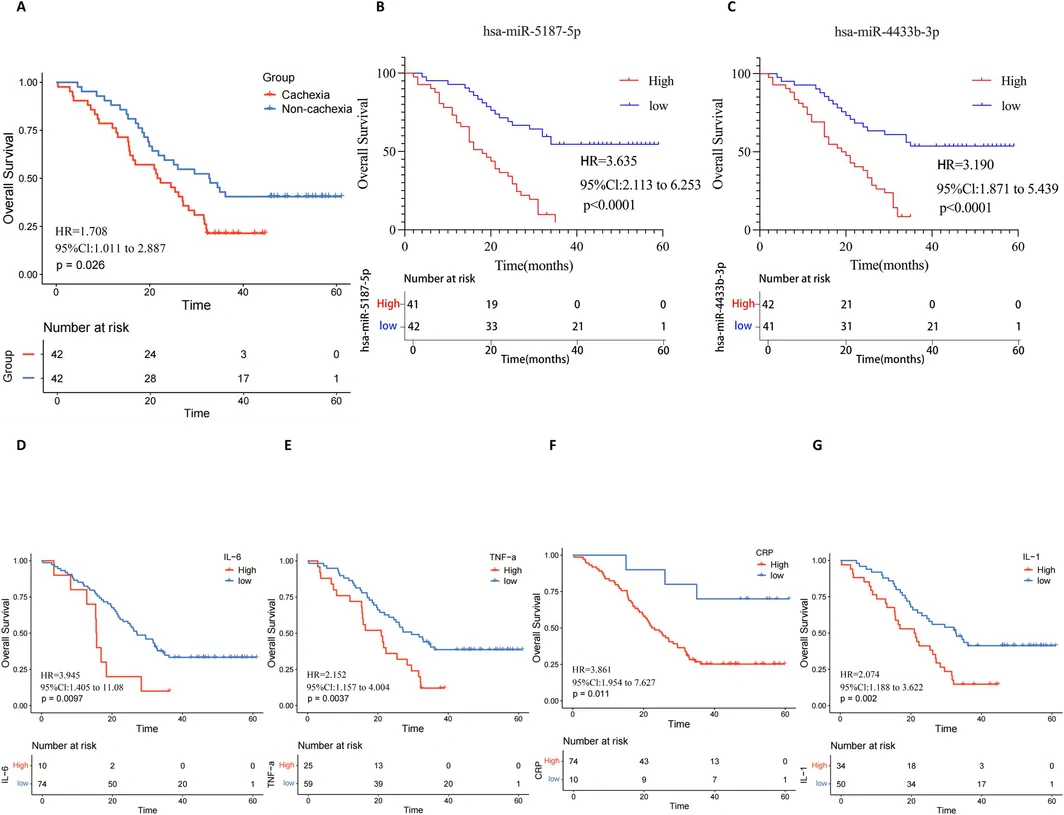
Unadjusted associations with overall survival in ESCC. Kaplan–Meier curves according to (A) cachexia status, (B) hsa‐miR‐5187‐5p, (C) hsa‐miR‐4433b‐3p, (D) IL‐6, (E) TNF‐α, (F) CRP and (G) IL‐1β. Hazard ratios were estimated using univariate Cox regression and were not adjusted for TNM stage, age, sex or other clinicopathological variables.

### 3.5 Circulating miRNAs and Inflammatory Markers Across the ESCC‐Cachexia Continuum

In univariate logistic regression (Table 2), neither hsa‐miR‐5187‐5p nor hsa‐miR‐4433b‐3p was associated with noncachectic ESCC versus healthy controls. Both were associated with cachectic ESCC versus controls (hsa‐miR‐5187‐5p: OR 1.104, 95% CI 1.056–1.170, *p* = 0.0001; hsa‐miR‐4433b‐3p: OR 1.089, 95% CI 1.048–1.147, *p* = 0.0002) and with cachectic versus noncachectic ESCC (hsa‐miR‐5187‐5p: OR 1.354, 95% CI 1.185–1.636, *p* = 0.0002; hsa‐miR‐4433b‐3p: OR 1.069, 95% CI 1.036–1.115, *p* = 0.0004). IL‐6 and IL‐1β were associated with all three group comparisons; TNF‐α was significant in controls versus noncachectic ESCC and noncachectic versus cachectic ESCC; CRP was significant in comparisons involving cachexia. Albumin was inversely associated with cachexia (controls vs. cachexia: OR 0.3553, *p* = 0.0004; noncachexia vs. cachexia: OR 0.4059, *p* < 0.0001).

**TABLE 2:** Univariate logistic regression analysis of miRNAs associated with ESCC and cachexia progression.

	Healthy control vs. ESCC without cachexia			Healthy control vs. ESCC without cachexia			ESCC without cachexia vs. ESCC with cachexia		
Variable	OR	95% CI	*p* value	OR	95% CI	*p* value	OR	95% CI	*p* value
miR‐5187‐5p	0.9763	0.8996–1.025	0.4145	1.104	1.056–1.170	0.0001	1.354	1.185–1.636	0.0002
miR‐4433b‐3p	1.010	0.9735–1.051	0.6111	1.089	1.048–1.147	0.0002	1.069	1.036–1.115	0.0004
IL‐6	72.97	10.06–6590	0.0048	23.511	2.625–210.584	0.005	3.043	1.826–5.596	< 0.0001
IL‐1	2.267	1.682–3.318	< 0.0001	19.72	3.706–780.1	0.0212	3.461	2.217–6.492	< 0.0001
CRP	1.092	1.034–1.290	0.0855	1.021	1.008–1.033	< 0.001	1.016	1.010–1.023	< 0.0001
TNF‐α	1.080	1.050–1.120	< 0.0001	1.420	0.973–2.073	0.069	1.136	1.082–1.220	< 0.0001
Albumin	0.8845	0.7661–1.012	0.0810	0.3553	0.1677–0.5511	0.0004	0.4059	0.2372–0.5749	< 0.0001

In exploratory multivariable models containing inflammatory markers and albumin (Table 3), hsa‐miR‐5187‐5p remained associated with cachectic ESCC versus healthy controls (OR 2.810, 95% CI 1.224–5.323; *p* = 0.006) and cachectic versus noncachectic ESCC (OR 12.885, 95% CI 1.180–51.410; *p* = 0.023) but not no‐cachectic ESCC versus controls (*p* = 0.433). hsa‐miR‐4433b‐3p was not significant in the corresponding adjusted models (all *p* > 0.05). Several inflammatory markers and albumin also remained associated with group status. Because TNM stage, age, sex and other potential confounders were not included and some estimates had wide CIs, these models should be interpreted as exploratory rather than evidence of independent biomarker performance.

**TABLE 3:** Multivariate logistic regression analysis of miRNAs in ESCC and cachexia progression.

	Healthy control vs. ESCC without cachexia					Healthy control vs. ESCC with cachexia					ESCC without cachexia vs. ESCC with cachexia				
Variable	*β*	Wald χ^2^	OR	95% CI	*p* value	*β*	Wald χ^2^	OR	95% CI	*p* value	*β*	Wald χ^2^	OR	95% CI	*p* value
Model 1															
miR‐5187‐5p	−0.353	0.614	0.703	0.306–1.711	0.433	1.033	7.502	2.810	1.224–5.323	0.006	2.556	5.145	12.885	1.180–51.410	0.023
IL‐6	4.837	50.963	126.063	21.585–273.804	< 0.001	2.993	54.233	19.946	8.104–36.864	< 0.001	0.310	0.232	1.363	0.526–5.851	0.630
IL‐1	1.699	13.004	5.466	2.353–13.587	< 0.001	2.872	58.920	17.671	7.061–28.032	< 0.001	1.595	6.002	4.926	1.791–19.619	0.014
CRP	4.356	54.900	77.972	17.813–155.705	< 0.001	2.937	227.916	18.854	11.736–25.025	< 0.001	1.396	5.129	4.037	1.311–15.375	0.024
TNF‐α	0.991	2.642	2.693	1.041–11.520	0.104	2.614	90.252	13.660	7.010–20.398	< 0.001	3.432	44.239	30.942	7.507–54.218	< 0.001
Albumin	1.214	1.805	3.367	0.302–7.062	0.179	−1.209	11.503	0.298	0.149–0.583	< 0.001	−2.900	16.494	0.055	0.019–0.249	< 0.001
Model 2															
miR‐4433b‐3p	−0.396	0.642	0.673	0.373–2.918	0.423	−0.055	0.021	0.946	0.701–3.556	0.885	0.023	0.000	1.023	0.657–119.398	0.985
IL‐6	4.784	49.427	119.588	19.873–234.667	< 0.001	3.075	46.268	21.647	7.913–40.736	< 0.001	−0.054	0.002	0.947	0.296–31.070	0.965
IL‐1	1.591	11.022	4.907	2.201–14.848	< 0.001	2.917	54.442	18.488	6.993–30.990	< 0.001	0.751	0.257	2.120	0.821–104.595	0.612
CRP	4.360	56.022	78.230	17.352–166.686	< 0.001	3.050	199.591	21.110	12.196–27.614	< 0.001	2.255	3.915	9.536	1.505–168.173	0.048
TNF‐α	1.187	3.991	3.276	1.249–12.467	0.046	2.739	79.502	15.478	7.692–23.732	< 0.001	9.087	14.533	8839.038	22.092–60434.117	< 0.001
Albumin	1.293	2.359	3.644	0.350–6.618	0.125	−1.219	11.395	0.296	0.143–0.529	< 0.001	−7.007	13.347	0.001	0.000–0.124	< 0.001

### 3.6 Associations With Inflammatory Markers and Albumin

Both candidate miRNAs showed significant but weak‐to‐moderate cross‐sectional correlations with inflammatory and biochemical markers (Figure 4). Correlations with IL‐6 were weak (hsa‐miR‐5187‐5p: r = 0.2706, *p* = 0.0026; hsa‐miR‐4433b‐3p: *r* = 0.2754, *p* = 0.0030). Positive correlations were also observed with TNF‐α (*r* = 0.3919 and 0.4733), IL‐1β (*r* = 0.4455 and 0.3629) and CRP (*r* = 0.3880 and 0.4099; all *p* < 0.0001). Albumin correlated inversely with hsa‐miR‐5187‐5p (*r* = −0.4183; *p* < 0.0001) and hsa‐miR‐4433b‐3p (*r* = −0.3446; *p* = 0.0002). These modest coefficients indicate partial covariation and do not demonstrate direct biological regulation or causality.

**FIGURE 4:**
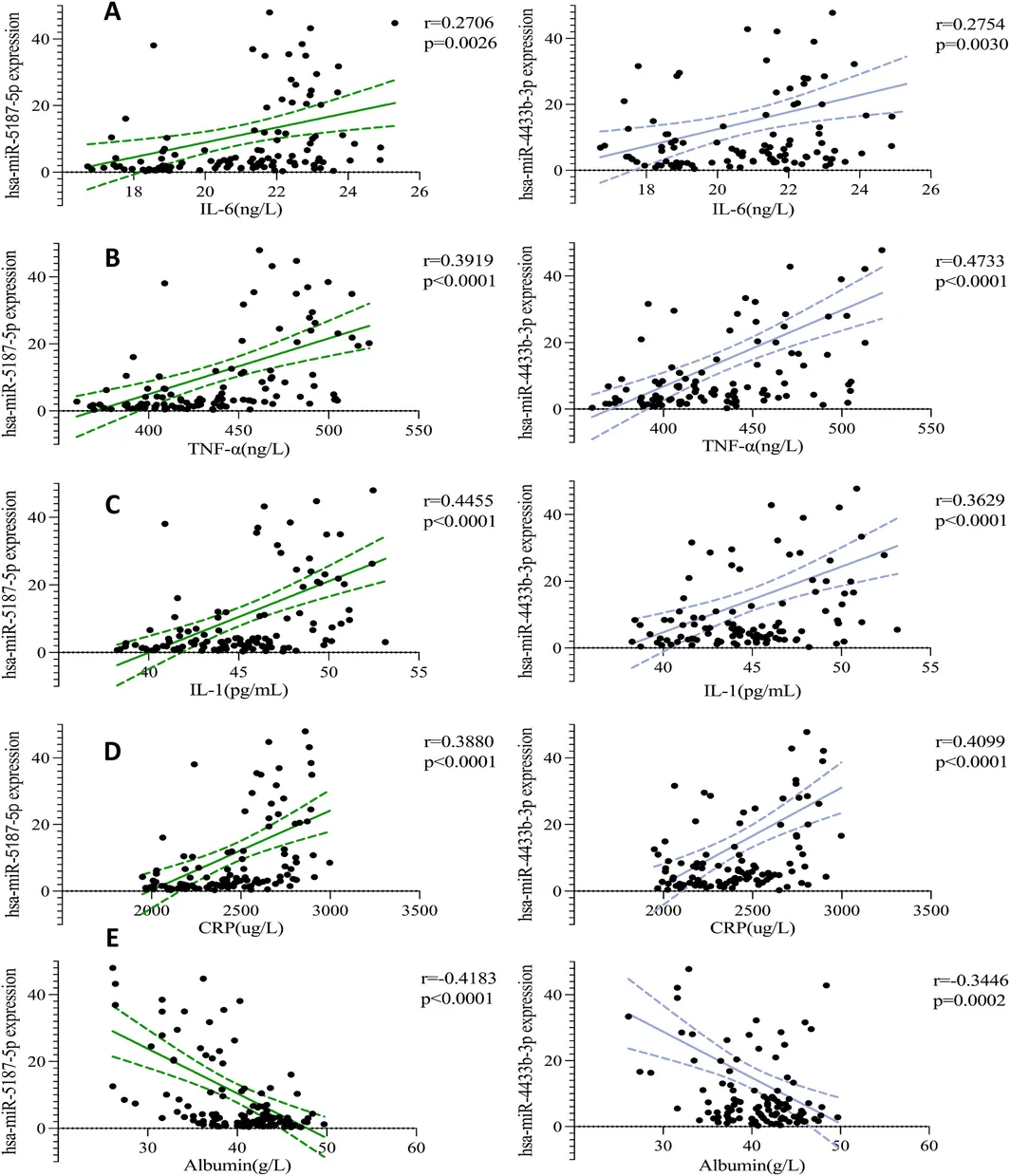
Associations of candidate miRNAs with inflammatory markers and albumin. Cross‐sectional correlations of hsa‐miR‐5187‐5p and hsa‐miR‐4433b‐3p with (A) IL‐6, (B) TNF‐α, (C) IL‐1β, (D) CRP and (E) albumin. Fitted lines and confidence bands are shown. Correlations were weak to moderate and do not establish causality.

### 3.7 Functional Enrichment and Pathway Network Analysis

GO and KEGG analyses were performed using the complete sets of differentially expressed miRNAs (Figure 5 and Tables S6 and S7). ESCC versus healthy‐control differences were enriched in signalling‐related biological processes and oncogenic, angiogenic and metabolic pathways, including VEGF, ErbB, p53, glycosphingolipid biosynthesis and amino‐acid metabolism. By contrast, cachectic versus noncachectic ESCC differences were enriched in lipid metabolism, oxidative stress, cellular senescence, inflammatory regulation, cytokine‐cytokine receptor interaction, JAK–STAT, PI3K‐Akt, TGF‐β and insulin signalling. Predicted target‐network integration linked hsa‐miR‐5187‐5p and hsa‐miR‐4433b‐3p to TNF/cytokine signalling, growth‐factor pathways and metabolic control (Figure 5E and Tables S8 and S9). Because these networks were based on predicted targets, they are hypothesis‐generating rather than evidence of direct miRNA‐mediated regulation.

**FIGURE 5:**
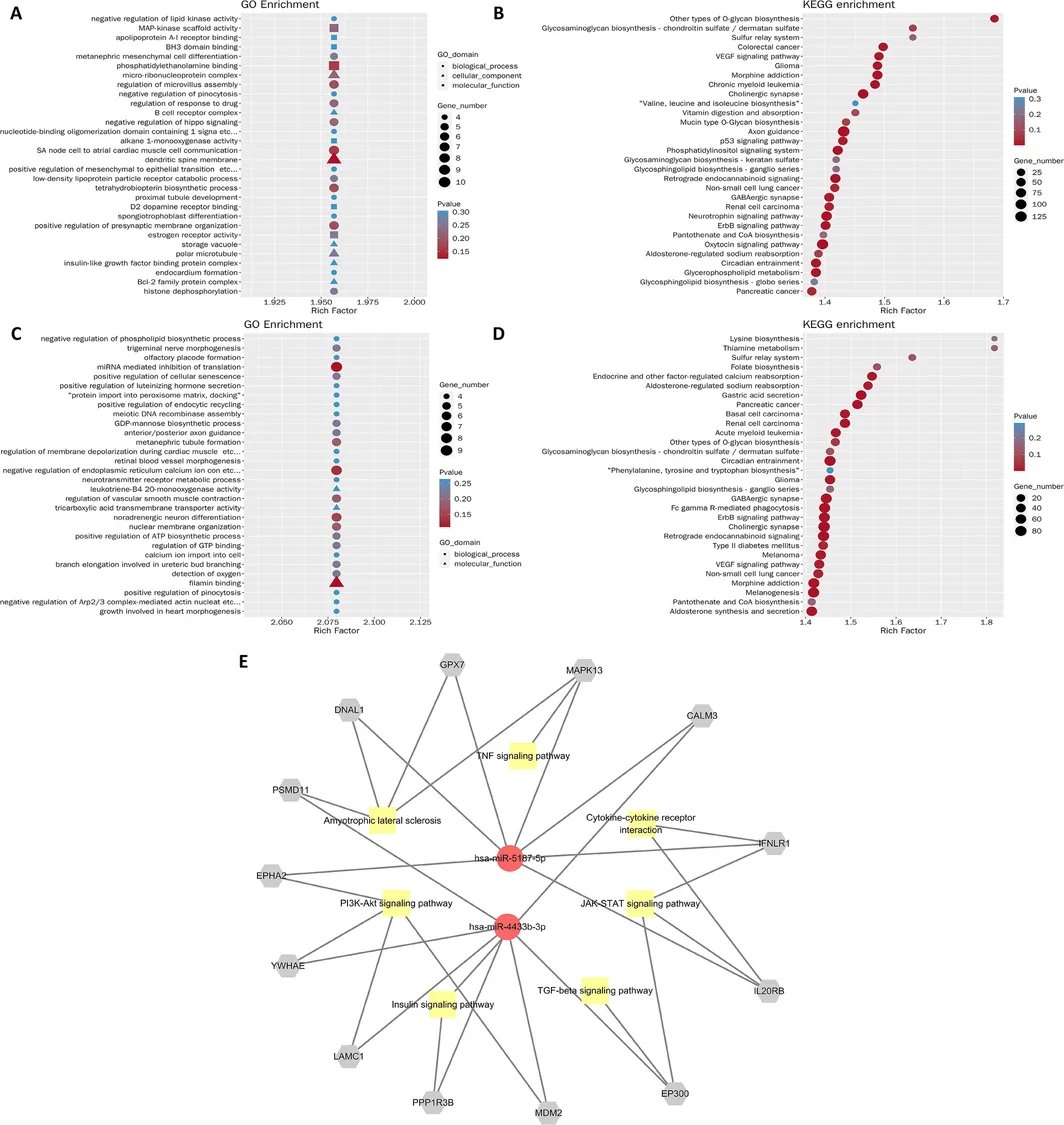
Functional enrichment and pathway network analysis. (A, B) GO and KEGG enrichment for miRNAs differentially expressed between ESCC and healthy controls. (C, D) GO and KEGG enrichment for cachectic versus noncachectic ESCC, highlighting inflammatory and metabolic pathways. (E) Predicted miRNA‐target gene‐pathway network for hsa‐miR‐5187‐5p and hsa‐miR‐4433b‐3p.

## 4 Discussion

The present study identifies hsa‐miR‐5187‐5p and hsa‐miR‐4433b‐3p as circulating miRNAs associated with ESCC‐related cachexia. Both increased stepwise across healthy controls, noncachectic ESCC and cachectic ESCC, and both showed stronger within‐cohort discrimination when cachectic ESCC was one of the comparison groups. Cachectic patients also had lower albumin, total protein and prealbumin and higher IL‐6, TNF‐α, CRP and IL‐1β, whereas the candidate miRNAs showed weak‐to‐moderate correlations with these inflammatory and biochemical markers. These results are consistent with the concept that ESCC cachexia reflects an integrated inflammatory, nutritional and metabolic phenotype rather than reduced intake alone, and they extend prior observations linking miRNA dysregulation to human cancer cachexia and muscle wasting [21, 22]. At the same time, the observational design, stage imbalance and lack of independent validation require a cautious interpretation: The identified miRNAs are best regarded as cachexia‐associated circulating indicators rather than established diagnostic or prognostic biomarkers.

This distinction is particularly important in ESCC because dysphagia can produce substantial calorie and protein deficiency independently of systemic catabolism [2, 23]. Nevertheless, cancer cachexia differs biologically from simple starvation. Persistent tumour‐host signalling can increase inflammatory cytokines, accelerate skeletal‐muscle proteolysis, promote adipose‐tissue loss, impair insulin action and reduce anabolic responsiveness [6, 24, 25]. In clinical practice, these processes can coexist with reduced intake and may help explain why nutritional support alone does not fully reverse established cachexia. Our findings of increased inflammatory markers and lower circulating nutritional proteins in cachectic ESCC are compatible with this combined nutritional‐inflammatory‐metabolic model. Because weight loss and BMI were components of the cachexia definition used in this study, however, their between‐group differences were expected by design. Similarly, estimated body fat and fat‐free mass indices were derived from BMI, age and sex and cannot be interpreted as direct measurements of tissue depletion.

The candidate miRNAs may reflect biological processes relevant to cachexia, although their mechanistic role remains unknown. Circulating miRNAs can arise from tumour cells, immune cells, skeletal muscle, adipose tissue or other organs and may be transported within extracellular vesicles or protein complexes [11, 12, 13, 26]. Experimental work has shown that tumour‐derived extracellular‐vesicle miRNAs can participate in tumour‐host communication and induce muscle injury; for example, miR‐21‐containing microvesicles can promote myoblast death through TLR7‐dependent signalling [16], and other cancer‐derived exosomal miRNAs have been implicated in muscle apoptosis [27]. These observations provide biological plausibility for circulating miRNAs as more than passive disease markers. However, the present study measured abundance in plasma only and did not determine the source, extracellular carrier, target tissue, direct mRNA targets or functional consequences of hsa‐miR‐5187‐5p or hsa‐miR‐4433b‐3p.

The enrichment analyses further linked cachexia‐associated miRNA changes to pathways that are well established in muscle wasting and systemic metabolic dysfunction. JAK–STAT signalling, particularly sustained IL‐6/STAT3 activation, is central to cachexia biology [28] and can drive ubiquitin‐proteasome activity, mitochondrial dysfunction and impaired myogenesis [29, 30]. Reduced PI3K‐Akt signalling contributes to impaired protein synthesis and insulin responsiveness [31], whereas TGF‐β signalling can promote fibrosis and defective muscle regeneration [32, 33, 34]. Enrichment of cytokine‐receptor, insulin and metabolic pathways therefore supports the biological relevance of the circulating miRNA signature. Nevertheless, GO/KEGG enrichment and predicted target networks cannot establish that either candidate directly regulates these pathways. These analyses should be regarded as hypothesis‐generating and require experimental validation in muscle and other relevant tissues.

The discriminatory analyses likewise require restraint. Although the AUCs for cachectic versus noncachectic ESCC were 0.8594 for hsa‐miR‐5187‐5p and 0.8175 for hsa‐miR‐4433b‐3p, candidate selection, qRT‐PCR assessment and ROC evaluation were performed within the same overall cohort. The reported cut‐offs, sensitivities and specificities therefore may overestimate performance in new patients. Moreover, cachectic patients had a higher proportion of advanced TNM‐stage disease. Tumour burden can independently increase systemic inflammation, worsen nutritional status, alter circulating miRNA profiles and shorten survival. TNM stage, age, sex and other clinicopathological variables were not systematically included in the ROC, correlation, survival or logistic‐regression analyses. Thus, apparent discrimination of cachexia may partly reflect disease burden. Independent, stage‐balanced validation is essential before either miRNA can be considered clinically useful.

The regression findings illustrate this limitation. hsa‐miR‐5187‐5p remained statistically associated with cachectic ESCC in selected multivariable logistic models containing inflammatory markers and albumin, whereas hsa‐miR‐4433b‐3p did not. However, the models were based on a modest sample and included several biologically correlated covariates, resulting in wide confidence intervals for some estimates. More importantly, they did not include TNM stage or other major clinicopathological confounders. Accordingly, statistical significance in these models should not be interpreted as independence from tumour burden. The same caveat applies to survival. Cachexia, both candidate miRNAs and inflammatory markers were associated with shorter overall survival in univariate analyses, but no multivariable Cox model was performed. The study therefore cannot determine whether the miRNAs provide prognostic information beyond tumour stage, age, sex or other established factors.

Several additional limitations should be emphasized. The cross‐sectional design precludes temporal or causal inference and cannot determine whether circulating miRNA alterations precede clinically evident cachexia. Direct body‐composition measurements were unavailable; equation‐derived fat and fat‐free mass indices cannot distinguish skeletal muscle from other lean tissues or quantify specific adipose depots. The single‐centre cohort was relatively small and lacked an external validation set. Circulating miRNA measurement is also sensitive to preanalytical conditions and normalization strategy [19, 20, 35]. U6 was used as the endogenous qRT‐PCR reference, but future work should evaluate multiple stable endogenous controls or other validated normalization approaches. Finally, the weak‐to‐moderate correlations with inflammatory markers indicate substantial unexplained variability and do not support direct causal relationships.

Future studies should use prospective, multicentre, stage‐balanced cohorts with separate discovery and validation sets, longitudinal blood sampling and direct assessment of skeletal muscle and adipose tissue by computed tomography, dual‐energy X‐ray absorptiometry or other validated methods. Adequately powered multivariable analyses should test whether circulating miRNAs add incremental information beyond TNM stage, inflammatory markers, nutritional measures and demographic factors. Serial measurements could determine whether miRNA changes track progression or response to nutritional, anti‐inflammatory or anticancer interventions. Functional experiments are also needed to identify the cellular origins and molecular targets of hsa‐miR‐5187‐5p and hsa‐miR‐4433b‐3p. Because timely recognition may facilitate supportive and nutritional intervention [36], independently validated circulating miRNAs could complement conventional cachexia assessment in ESCC by providing a minimally invasive readout of the accompanying inflammatory‐metabolic milieu.

## 5 Conclusion

Circulating hsa‐miR‐5187‐5p and hsa‐miR‐4433b‐3p were associated with ESCC‐related cachexia, systemic inflammation, impaired biochemical nutritional indices and shorter unadjusted survival. Their within‐cohort discrimination is promising but exploratory. Independent validation, direct body‐composition assessment, longitudinal sampling and adjustment for tumour stage and other clinicopathological factors are required before diagnostic, prognostic or clinical utility can be established.

## Funding

This work was supported by the National Natural Science Foundation of China (82103021) and the China Postdoctoral Science Foundation (2022M721725), both awarded to S. Ding; the Nantong First People's Hospital Provincial and Ministerial Level High‐level Science and Technology Project Cultivation Fund (YPYJJYB24015), awarded to Y. Li; the ‘14th Five‐Year Plan’ Nantong Clinical Medical Center (Innovation Platform) and the Youth Medical Expert Training Program of the Nantong Municipal Health Commission.

## Conflicts of Interest

The authors declare no conflicts of interest.

## Supporting information


**Table S1:** RNA quality and PCR efficiency for all samples.

**Table S2:** Oligonucleotide sequences for miRNA RT‐qPCR.

**Table S3:** Normalized miRNA levels were compared pairwise among healthy control (HC), non‐cachexia, and cachexia.

**Table S4:** Differentially expressed miRNAs identified by sequencing between healthy controls and cachexia.

**Table S5:** Differentially expressed miRNAs identified by sequencing between healthy controls and non‐cachexia.

**Table S6:** Genes significantly targeted by differentially expressed miRNAs identified by sequencing between healthy controls and cachexia.

**Table S7:** Genes significantly targeted by differentially expressed miRNAs identified by sequencing between healthy controls and Non‐cachexia.

**Table S8:** miRTarBase enrichment of two genes of interest.

**Table S9:** Pathway‐gene interactions included in the network.

**Figure S1: Elevated circulating inflammatory markers in ESCC patients with cachexia**. Box‐and‐whisker plots showing serum concentrations of (A) IL‐6, (B) TNF‐α, (C) IL‐1β, and (D) CRP in healthy controls (n=42), ESCC patients without cachexia (non‐cachexia) (n=42), and ESCC patients with cachexia (n=42). Data are presented as median with interquartile range; whiskers indicate minimum and maximum values. Statistical significance was assessed using appropriate multiple‐group comparison tests. ****P < 0.0001.

## Data Availability

The data that support the findings of this study are available on request from the corresponding author. The data are not publicly available due to privacy or ethical restrictions.
