# Supplementary material for: Circulating MicroRNAs Reflect Systemic Inflammation and Metabolic Dysfunction in Oesophageal Squamous Cell Carcinoma–Associated Cachexia
Source: J Cachexia Sarcopenia Muscle. 2026 Oct 22;17(5):e70409. doi: 10.1002/jcsm.70409 (PMC13688428; doi:10.1002/jcsm.70409)
Supplement: Supplementary file 1 [file JCSM-17-e70409-s001.docx]

**Table S1: RNA quality and PCR efficiency for all samples.**

|  | **RNA quality** | **Individual PCR efficiency** | |
| --- | --- | --- | --- |
| **Sample** | **A260/280** | **miR-4433b-3p** | **miR-5187-5p** |
| N_1 | 1.93 | 1.812 | 1.742 |
| N_2 | 1.81 | 1.734 | 1.885 |
| N_3 | 1.86 | 1.889 | 1.813 |
| N_4 | 1.84 | 1.765 | 1.769 |
| N_5 | 1.95 | 1.821 | 1.858 |
| N_6 | 1.94 | 1.903 | 1.724 |
| N_7 | 1.98 | 1.776 | 1.891 |
| N_8 | 1.82 | 1.858 | 1.806 |
| N_9 | 1.88 | 1.742 | 1.777 |
| N_10 | 1.81 | 1.817 | 1.834 |
| N_11 | 1.80 | 1.791 | 1.795 |
| N_12 | 1.96 | 1.864 | 1.712 |
| N_13 | 1.96 | 1.829 | 1.879 |
| N_14 | 1.82 | 1.705 | 1.846 |
| N_15 | 1.87 | 1.748 | 1.731 |
| N_16 | 1.88 | 1.882 | 1.822 |
| N_17 | 1.94 | 1.796 | 1.864 |
| N_18 | 1.85 | 1.833 | 1.756 |
| N_19 | 1.99 | 1.759 | 1.893 |
| N_20 | 1.97 | 1.871 | 1.831 |
| N_21 | 1.83 | 1.724 | 1.778 |
| N_22 | 1.87 | 1.855 | 1.857 |
| N_23 | 1.84 | 1.813 | 1.741 |
| N_24 | 1.93 | 1.784 | 1.818 |
| N_25 | 1.99 | 1.897 | 1.789 |
| N_26 | 1.90 | 1.768 | 1.865 |
| N_27 | 1.82 | 1.739 | 1.724 |
| N_28 | 1.82 | 1.826 | 1.898 |
| N_29 | 1.98 | 1.861 | 1.807 |
| N_30 | 1.94 | 1.792 | 1.768 |
| N_31 | 1.96 | 1.744 | 1.841 |
| N_32 | 1.84 | 1.879 | 1.772 |
| N_33 | 1.99 | 1.808 | 1.885 |
| N_34 | 1.86 | 1.766 | 1.826 |
| N_35 | 1.95 | 1.835 | 1.763 |
| N_36 | 1.89 | 1.723 | 1.835 |
| N_37 | 1.87 | 1.892 | 1.794 |
| N_38 | 1.97 | 1.851 | 1.709 |
| N_39 | 1.88 | 1.777 | 1.881 |
| N_40 | 1.88 | 1.804 | 1.849 |
| N_41 | 1.99 | 1.738 | 1.739 |
| N_42 | 1.89 | 1.865 | 1.821 |
| E_1 | 1.81 | 1.791 | 1.866 |
| E_2 | 1.94 | 1.828 | 1.755 |
| E_3 | 1.86 | 1.753 | 1.892 |
| E_4 | 1.90 | 1.883 | 1.832 |
| E_5 | 1.95 | 1.812 | 1.777 |
| E_6 | 1.94 | 1.769 | 1.856 |
| E_7 | 1.98 | 1.846 | 1.741 |
| E_8 | 1.90 | 1.734 | 1.819 |
| E_9 | 1.91 | 1.895 | 1.784 |
| E_10 | 1.96 | 1.821 | 1.865 |
| E_11 | 1.89 | 1.758 | 1.731 |
| E_12 | 1.83 | 1.872 | 1.896 |
| E_13 | 1.96 | 1.806 | 1.808 |
| E_14 | 1.93 | 1.741 | 1.759 |
| E_15 | 1.87 | 1.889 | 1.842 |
| E_16 | 1.97 | 1.827 | 1.773 |
| E_17 | 1.96 | 1.764 | 1.887 |
| E_18 | 1.84 | 1.851 | 1.825 |
| E_19 | 1.93 | 1.793 | 1.761 |
| E_20 | 1.96 | 1.709 | 1.834 |
| E_21 | 1.88 | 1.836 | 1.792 |
| E_22 | 1.88 | 1.878 | 1.718 |
| E_23 | 1.96 | 1.722 | 1.879 |
| E_24 | 1.93 | 1.809 | 1.847 |
| E_25 | 1.99 | 1.867 | 1.736 |
| E_26 | 1.86 | 1.754 | 1.821 |
| E_27 | 1.93 | 1.891 | 1.863 |
| E_28 | 1.96 | 1.833 | 1.755 |
| E_29 | 1.82 | 1.776 | 1.894 |
| E_30 | 1.86 | 1.858 | 1.832 |
| E_31 | 1.83 | 1.742 | 1.777 |
| E_32 | 1.83 | 1.819 | 1.856 |
| E_33 | 1.94 | 1.784 | 1.741 |
| E_34 | 1.97 | 1.865 | 1.818 |
| E_35 | 1.90 | 1.731 | 1.789 |
| E_36 | 1.96 | 1.896 | 1.864 |
| E_37 | 1.83 | 1.808 | 1.724 |
| E_38 | 1.93 | 1.759 | 1.897 |
| E_39 | 1.86 | 1.842 | 1.807 |
| E_40 | 1.82 | 1.773 | 1.768 |
| E_41 | 1.81 | 1.887 | 1.841 |
| E_42 | 1.89 | 1.825 | 1.772 |
| C_1 | 1.88 | 1.761 | 1.886 |
| C_2 | 1.97 | 1.834 | 1.826 |
| C_3 | 1.95 | 1.792 | 1.763 |
| C_4 | 1.84 | 1.718 | 1.835 |
| C_5 | 1.89 | 1.879 | 1.794 |
| C_6 | 1.95 | 1.847 | 1.712 |
| C_7 | 1.87 | 1.736 | 1.882 |
| C_8 | 1.88 | 1.821 | 1.849 |
| C_9 | 1.97 | 1.863 | 1.739 |
| C_10 | 1.83 | 1.755 | 1.822 |
| C_11 | 1.96 | 1.894 | 1.866 |
| C_12 | 1.98 | 1.832 | 1.756 |
| C_13 | 1.96 | 1.777 | 1.893 |
| C_14 | 1.93 | 1.856 | 1.831 |
| C_15 | 1.98 | 1.741 | 1.778 |
| C_16 | 1.86 | 1.818 | 1.857 |
| C_17 | 1.97 | 1.789 | 1.742 |
| C_18 | 1.87 | 1.864 | 1.818 |
| C_19 | 1.98 | 1.724 | 1.791 |
| C_20 | 1.86 | 1.898 | 1.864 |
| C_21 | 1.88 | 1.807 | 1.725 |
| C_22 | 1.99 | 1.768 | 1.899 |
| C_23 | 1.98 | 1.841 | 1.806 |
| C_24 | 1.83 | 1.772 | 1.769 |
| C_25 | 1.97 | 1.885 | 1.842 |
| C_26 | 1.83 | 1.826 | 1.774 |
| C_27 | 1.93 | 1.763 | 1.888 |
| C_28 | 1.90 | 1.835 | 1.824 |
| C_29 | 1.93 | 1.794 | 1.764 |
| C_30 | 1.92 | 1.712 | 1.836 |
| C_31 | 1.96 | 1.881 | 1.793 |
| C_32 | 1.91 | 1.849 | 1.713 |
| C_33 | 1.92 | 1.739 | 1.883 |
| C_34 | 1.96 | 1.822 | 1.848 |
| C_35 | 1.95 | 1.866 | 1.738 |
| C_36 | 1.91 | 1.756 | 1.823 |
| C_37 | 1.99 | 1.893 | 1.865 |
| C_38 | 1.88 | 1.831 | 1.757 |
| C_39 | 1.91 | 1.778 | 1.892 |
| C_40 | 1.97 | 1.857 | 1.833 |
| C_41 | 1.92 | 1.812 | 1.779 |
| C_42 | 1.97 | 1.734 | 1.858 |

***RNA quality (A260/280) was assessed using the ND-1000 Nanodrop spectrophotometer. PCR efficiency was determined for each sample and primer using LinRegPCR software.***
