# Supplementary material for: Circulating MicroRNAs Reflect Systemic Inflammation and Metabolic Dysfunction in Oesophageal Squamous Cell Carcinoma–Associated Cachexia
Source: J Cachexia Sarcopenia Muscle. 2026 Oct 22;17(5):e70409. doi: 10.1002/jcsm.70409 (PMC13688428; doi:10.1002/jcsm.70409)
Supplement: Supplementary file 2 [file JCSM-17-e70409-s003.docx]

**Table S3: Normalized miRNA levels were compared pairwise among healthy control(HC), non-cachexia, and cachexia.**

| **miRNA** | **HC vs non-cachexia**  **(2-ΔΔCt)** | **P-value** | **HC vs cachexia**  **(2-ΔΔCt)** | **P-value** | **non-cachexia vs cachexia**  **(2-ΔΔCt)** | **P-value** |
| --- | --- | --- | --- | --- | --- | --- |
| hsa- miR-4433b-3p | 1.48 | 0.0321 | 148.21 | 0.0216 | 99.86 | 0.0220 |
| hsa-miR-5187-5p | 1.30 | 0.0431 | 12.48 | <0.0001 | 9.60 | <0.0001 |

**HC vs non-cachexia, normalized to HC. HC vs cachexia, normalized to HC. Non-cachexia vs cachexia, normalized to non-cachexia.**
