# Supplementary material for: Circulating MicroRNAs Reflect Systemic Inflammation and Metabolic Dysfunction in Oesophageal Squamous Cell Carcinoma–Associated Cachexia
Source: J Cachexia Sarcopenia Muscle. 2026 Oct 22;17(5):e70409. doi: 10.1002/jcsm.70409 (PMC13688428; doi:10.1002/jcsm.70409)
Supplement: Supplementary file 3 [file JCSM-17-e70409-s004.docx]

**Figure S1. Elevated circulating inflammatory markers in ESCC patients with cachexia.**

Box-and-whisker plots showing serum concentrations of (A) IL-6, (B) TNF-α, (C) IL-1β, and (D) CRP in healthy controls (n=42), ESCC patients without cachexia (non-cachexia) (n=42), and ESCC patients with cachexia (n=42). Data are presented as median with interquartile range; whiskers indicate minimum and maximum values. Statistical significance was assessed using appropriate multiple-group comparison tests. ****P < 0.0001.
