# Supplementary material for: Circulating MicroRNAs Reflect Systemic Inflammation and Metabolic Dysfunction in Oesophageal Squamous Cell Carcinoma–Associated Cachexia
Source: J Cachexia Sarcopenia Muscle. 2026 Oct 22;17(5):e70409. doi: 10.1002/jcsm.70409 (PMC13688428; doi:10.1002/jcsm.70409)
Supplement: Supplementary file 4 [file JCSM-17-e70409-s005.docx]

**Table S2:** **Oligonucleotide sequences for miRNA RT-qPCR.**

| **miRNA name** | **Oligonucleotide sequence (5′–3′)** | **Company** |
| --- | --- | --- |
| hsa-miR-4433b-3p | AGTGGGGGGTGGGACGTAAA | Sangon Biotech |
| hsa-miR-5187-5p | ACACTCCAGCTGGGTGGGATGAGGGATTGA | Sangon Biotech |
