# Supplementary material for: Circulating MicroRNAs Reflect Systemic Inflammation and Metabolic Dysfunction in Oesophageal Squamous Cell Carcinoma–Associated Cachexia
Source: J Cachexia Sarcopenia Muscle. 2026 Oct 22;17(5):e70409. doi: 10.1002/jcsm.70409 (PMC13688428; doi:10.1002/jcsm.70409)
Supplement: Supplementary file 5 [file JCSM-17-e70409-s010.docx]

**Table S9: Pathway-gene interactions included in the network.**

| **miRNA** | **Pathways** | **Genes** | **Source** |
| --- | --- | --- | --- |
| hsa-miR-5187-5p | Amyotrophic lateral sclerosis | DNAL1 | miRTarBase |
| hsa-miR-5187-5p | Amyotrophic lateral sclerosis | GPX7 | miRTarBase |
| hsa-miR-5187-5p | Amyotrophic lateral sclerosis | MAPK13 | miRTarBase |
| hsa-miR-4433b-3p | Amyotrophic lateral sclerosis | PSMD11 | miRTarBase |
| hsa-miR-5187-5p | Cytokine-cytokine receptor interaction | IFNLR1 | miRTarBase |
| hsa-miR-5187-5p | Cytokine-cytokine receptor interaction | IL20RB | miRTarBase |
| hsa-miR-5187-5p | Insulin signaling pathway | CALM3 | miRTarBase |
| hsa-miR-4433b-3p | Insulin signaling pathway | PPP1R3B | miRTarBase |
| hsa-miR-4433b-3p | JAK-STAT signaling pathway | EP300 | miRTarBase |
| hsa-miR-5187-5p | JAK-STAT signaling pathway | IFNLR1 | miRTarBase |
| hsa-miR-5187-5p | JAK-STAT signaling pathway | IL20RB | miRTarBase |
| hsa-miR-5187-5p | PI3K-Akt signaling pathway | EPHA2 | miRTarBase |
| hsa-miR-4433b-3p | PI3K-Akt signaling pathway | YWHAE | miRTarBase |
| hsa-miR-4433b-3p | PI3K-Akt signaling pathway | MDM2 | miRTarBase |
| hsa-miR-4433b-3p | PI3K-Akt signaling pathway | LAMC1 | miRTarBase |
| hsa-miR-4433b-3p | TGF-beta signaling pathway | EP300 | miRTarBase |
| hsa-miR-5187-5p | TNF signaling pathway | MAPK13 | miRTarBase |
